# Identification of Glyceraldehyde-3-phosphate dehydrogenase (GAPDH) as a binding protein for a 68-kDa *Bacillus thuringiensis *parasporal protein cytotoxic against leukaemic cells

**DOI:** 10.1186/1423-0127-17-86

**Published:** 2010-11-13

**Authors:** Kanakeswary Krishnan, Jeremy Er An Ker, Shar Mariam Mohammed, Vishna Devi Nadarajah

**Affiliations:** 1 Department of Pharmacy, Faculty of Medicine and Health Sciences, International Medical University, No 126 Jalan 19/155B Bukit Jalil, Kuala Lumpur, 57000 Malaysia; 2 School of Postgraduate Studies, Faculty of Medicine and Health Sciences, International Medical University, No 126 Jalan 19/155B Bukit Jalil, Kuala Lumpur, 57000 Malaysia; 3 Department of Human Biology, Faculty of Medicine and Health Sciences, International Medical University, No 126 Jalan 19/155B Bukit Jalil, Kuala Lumpur, 57000 Malaysia

## Abstract

**Background:**

*Bacillus thuringiensis *(Bt), an ubiquitous gram-positive spore-forming bacterium forms parasporal proteins during the stationary phase of its growth. Recent findings of selective human cancer cell-killing activity in non-insecticidal Bt isolates resulted in a new category of Bt parasporal protein called parasporin. However, little is known about the receptor molecules that bind parasporins and the mechanism of anti-cancer activity. A Malaysian Bt isolate, designated Bt18 produces parasporal protein that exhibit preferential cytotoxic activity for human leukaemic T cells (CEM-SS) but is non-cytotoxic to normal T cells or other cancer cell lines such as human cervical cancer (HeLa), human breast cancer (MCF-7) and colon cancer (HT-29) suggesting properties similar to parasporin. In this study we aim to identify the binding protein for Bt18 in human leukaemic T cells.

**Methods:**

Bt18 parasporal protein was separated using Mono Q anion exchange column attached to a HPLC system and antibody was raised against the purified 68-kDa parasporal protein. Receptor binding assay was used to detect the binding protein for Bt18 parasporal protein in CEM-SS cells and the identified protein was sent for N-terminal sequencing. NCBI protein BLAST was used to analyse the protein sequence. Double immunofluorescence staining techniques was applied to localise Bt18 and binding protein on CEM-SS cell.

**Results:**

Anion exchange separation of Bt18 parasporal protein yielded a 68-kDa parasporal protein with specific cytotoxic activity. Polyclonal IgG (anti-Bt18) for the 68-kDa parasporal protein was successfully raised and purified. Receptor binding assay showed that Bt18 parasporal protein bound to a 36-kDa protein from the CEM-SS cells lysate. N-terminal amino acid sequence of the 36-kDa protein was GKVKVGVNGFGRIGG. NCBI protein BLAST revealed that the binding protein was Glyceraldehyde-3-phosphate dehydrogenase (GAPDH). Double immunofluorescence staining showed co-localisation of Bt18 and GAPDH on the plasma membrane of the CEM-SS cells.

**Conclusions:**

GAPDH has been well known as a glycolytic enzyme, but recently GAPDH was discovered to have roles in apoptosis and carcinogenesis. Pre-incubation of anti-GAPDH antibody with CEM-SS cells decreases binding of Bt18 to the susceptible cells. Based on a qualitative analysis of the immunoblot and immunofluorescence results, GAPDH was identified as a binding protein on the plasma membrane of CEM-SS cells for Bt18 parasporal protein.

## Background

*Bacillus thuringiensis *(Bt) was initially characterised as an insect pathogen, and its insecticidal activity was attributed largely to parasporal proteins. Recent studies, however, have reported that non-insecticidal Bt strains are more widely distributed than insecticidal ones [1]. This raises the question of whether non-insecticidal parasporal proteins have any biological activity which is as yet undiscovered.

In a pioneering study, it was reported that selective human cancer cell-killing activity is associated with some non-insecticidal Bt isolates resulting in a new category of Bt parasporal protein called parasporin. Parasporins are defined as bacterial parasporal proteins that are capable of preferentially killing cancer cells [2,3]. Mizuki *et al.*, (2000) obtained the first parasporin by expressing the *cry *gene encoding the Cry31Aa protein (also known as parasporin-1), which exhibits strong cytotoxicity against human leukemic T cells (MOLT-4), but did not exhibit insecticidal or hemolytic activities [4]. This was followed by the identification of three more proteins, Cry46Aa (parasporin-2), Cry41Aa (parasporin-3) and Cry45Aa (parasporin-4) also with selective cytotoxic activities against cancer cells [5-7]. Recently two more parasporin (PS5Aa1 and PS6Aa1) were added in the parasporin nomenclature [8]. Interestingly, a Malaysian Bt isolate, designated Bt18 produces parasporal protein that exhibit cytotoxic activity preferentially for human leukaemic T cells (CEM-SS) but is non-cytotoxic to normal T cells or other cancer cell lines such as HeLa, MCF-7 and HT-29 [9]. It was reported that Bt18 parasporal protein is cytotoxic to CEM-SS as 84% cell death was observed at 0.5 μg/mL (CD_50 _value of 0.1224 ± 0.0092 μg/mL) [9]. Bt18 produces parasporal protein, which is also non-hemolytic to human or rat erythrocytes after trypsin activation, shows therapeutic and diagnostic potential with regards to leukaemia. This finding has triggered interest in elucidating the mode of action of Bt18 parasporal protein. Questions arise on how Bt18 parasporal protein specifically recognise leukaemic T cells. Insecticidal Bt parasporal proteins are known to bind receptors on the insect brush border membrane and it is suggested that these receptors play a role in the specificity of insecticidal activity [10,11].

We hypothesise that Bt18 cell killing activity is receptor mediated in that Bt18 parasporal protein binds specifically to a binding protein on the plasma membrane. To identify the binding protein, qualitative analysis were performed on Bt18 and CEM-SS cells using immunoblot and immunofluorescent staining. Glyceraldehyde-3-phosphate dehydrogenase (GAPDH) was identified as a binding protein for Bt18.

## Methods

### Bacterial strains and growth conditions

The Bt isolates used in this study were from Institute for Medical Research (IMR), Malaysia. Bt collections and the subtypes were determined using H antigen serotyping. Bt isolates used were Bt18, *Bacillus thuringiensis *2 (Bt2), and *Bacillus thuringiensis *subsp *jegathesan *(Btj). The Bt isolates were cultured in nutrient broth supplemented with CaCl_2 _(0.01%), MgCl_2 _(0.08%) and MnCl_2 _(0.07%) at 30°C until more than 95% sporulation occurred.

### Preparation of spore-crystal mixture

Sporulated Bt cultures was treated with 1 M NaCl to osmotically lyse the bacterium to release the spore and crystals. The spore-crystal mixture was harvested by centrifugation at 13,000 *g *for 5 minutes, washed once with NaCl and twice with ice-cold water. The spore-crystal mixture was resuspended in Tris/KCl buffer (pH 7.5) before storing at -20°C.

The parasporal protein was separated from spores by ultracentrifugation of the spore-crystal mixture at 25000 *g*, 4°C for 16 hours on a discontinuous sucrose density gradient of 67, 72 and 79% (w/v) in Tris/KCl buffer (pH 7.5) [12].

### Solubilisation and activation of parasporal protein

The parasporal protein was solubilised in sodium carbonate buffer (pH 10.5) containing 10 mM DTT and activated with trypsin (1 mg/mL) for 1 hour at 37°C. The activated parasporal protein was desalted and concentrated using Amicon^® ^Ultra-4 centrifugal filter (Milipore). Protein concentration was estimated using the method of Bradford [13] using bovine serum albumin (BSA) as standard.

### Separation of Bt18 parasporal protein

The trypsin activated parasporal protein was separated using Mono Q anion exchange column attached to a HPLC system (Perkin Elmer Series 200). The column was pre-equilibrated in 20 mM Piperazine buffer (pH 9.8). Bound proteins were eluted with 0-1 M NaCl solution and monitored at 280 nm. The eluted protein were analysed by SDS-PAGE and desalted using Amicon^® ^Ultra-4 centrifugal filter (Milipore).

### Polyclonal antibody production

The parasporal protein was separated by SDS-PAGE and the 68-kDa protein was eluted from the gel by passive elution, overnight in elution buffer (pH 7.5). Eluted protein was concentrated and desalted using Amicon^® ^Ultra-4 centrifugal filter. 100 μg/mL of the protein were mixed with Freund's complete adjuvant and injected subcutaneously into 2 New Zealand White rabbits. Three booster immunisations with incomplete Freunds adjuvant were administered at 7 days, 28 days, and 42 days after primary immunisation. The ability of the antibody developed against the 68-kDa protein was determined by immunoblots. The anti-Bt18 antibody was purified using Melon Gel IgG purification kit (Pierce). Gravity-flow column procedure for antibody purification was used. Purified antibody was examined for purity by SDS-PAGE and protein concentration of IgG was determined by method of Bradford [13].

### Immunoblot assay to detect the binding of anti-Bt18 antibody to Bt18 parasporal protein

Solubilised and activated Bt18 parasporal protein was separated by SDS-PAGE and transferred to a nitrocellulose membrane. The membrane was blocked in 5% BSA in TBS, pH7.4 solution for 1 hour at room temperature. The primary antibody anti-Bt18 antibody (1:1000) was added and incubated for 1 hour at 37°C. The membrane was washed 5 × 5 minutes washes with TBS (pH 7.4) and incubated with secondary antibody HRP labeled (1:5000) for 1 hour at room temperature. After washing 5 × 5 minutes washes with TBS (pH 7.4), the membrane was developed with 4-chloro-1-naphtol substrate.

### Culture of human T4-lymphoblastoid (CEM-SS) cell line

CEM-SS cells were grown at 37°C with 5% CO_2 _in 75 mL tissue culture flask (Nunc) in RPMI 1640 supplemented with 10% fetal bovine serum, 0.1% sodium pyruvate, 0.1% HEPES, 0.1% glutamine and 1% penicillin-streptomycin.

### Detection of Bt18 parasporal protein in CEM-SS cells via immunostaining

CEM-SS cells (10^6^cells/mL) were harvested by centrifugation at 130 g for 5 minutes and washed 3 times in phosphate buffered saline (PBS) (pH 7.4). The washed cells were fixed using 4% paraformaldehyde for 10 minutes and smears were prepared on poly-L-lysine coated slides. The smears were dried overnight at room temperature. The smear was covered with 0.1% H_2_O_2 _in PBS for 10 minutes to quench endogenous peroxidase activity and rinsed 3 times with PBS. Non-specific binding was blocked using 10% BSA in PBS for 20 minutes. After 3 times washing in PBS, the smear was incubated with 100 μg/mL of trypsin activated Bt18 parasporal protein for 1 hour. The smear was washed 3 times in PBS and primary antibody (anti-Bt18 antibody, 1:1000) was added and incubated for 1 hour at room temperature. Then the smear was washed 3 times and incubated with secondary antibody horseradish peroxidase (HRP) labeled (1: 5000) for 45 minutes at room temperature. The smear was washed 3 times with PBS and rinsed with 0.5% triton X-100 in PBS for 30 seconds. Freshly prepared liquid DAB substrate solution (DakoCytomation) was incubated for 5 minutes. After rinsing with distilled water, the smear was counterstained with haematoxylin for 3 seconds.

### Detection of Bt18 binding protein using toxin overlay blot

To identify the Bt18 binding protein, two methods were used to prepare the cell binding protein containing sample. In one of the method, membrane proteins were prepared from CEM-SS cells by using Mem-PER Eukaryotic Membrane Protein Extraction Reagent Kit (Pierce). In the second method, CEM-SS cells lysate was prepared by sonication method. The membrane proteins and CEM-SS cells lysate were separated by SDS-PAGE and electrophoretically transferred to a nitrocellulose membrane. Membrane was blocked with 5% (w/v) bovine serum albumin (BSA) in tris buffered saline TBS (pH 7.4) for 2 hours at room temperature. This was followed by overnight incubation with 250 μg/mL of Bt18 parasporal protein at 4°C. Excess toxin was removed by 5 × 5 minutes washes with TBS (pH 7.4). The blot was then incubated with anti-Bt18 antibody (1:1000) for 1 hr at room temperature. The membrane was washed 5 × 5 minutes washes with TBS (pH 7.4) and incubated with secondary antibody HRP labeled (1:5000) for 1 hour at room temperature. After washing 5 × 5 minutes washes with TBS (pH 7.4), the membrane was developed with 4-chloro-1-naphtol substrate.

### N-terminal protein sequencing of binding protein

The identified binding protein was separated by SDS-PAGE and transferred to a PVDF membrane, stained with 0.1% Coomassie stain in 50% methanol, 7% acetic acid for 2 minutes. The membrane was de-stained in 50% methanol, 7% acetic acid, for 10 minutes. To completely de-stain the background, the membrane was incubated in 90% methanol, 10% acetic acid for 10 minutes. The membrane was sent to Vivantis Technologies-Biomolecular Research Facility-Newcastle Protein, Applied Biosystem - PROCISE (University of Newcastle, Australia) for N-terminal protein sequencing. The 15 amino acid sequence was analysed using the Basic Local Alignment Search Tool (BLAST), at the National Center for Biotechnology Information (NCBI) website http://blast.ncbi.nlm.nih.gov/Blast.cgi.

### Detection of GAPDH in CEM-SS cell lysate via immunoblot assay

CEM-SS cell lysate was prepared by sonication method and separated by SDS-PAGE. The separated cell lysate was transferred to a nitrocellulose membrane using Mini Trans-Blot Electrophoretic transfer cell (Bio-Rad). Membrane was blocked with 5% (w/v) bovine serum albumin (BSA) in tris buffered saline TBS (pH 7.4) for 2 hours at room temperature. The membrane was then incubated with anti-GAPDH antibody (1:2000) for 1 hr at room temperature. The membrane was washed 5 × 5 minutes washes with TBS (pH 7.4) and incubated with secondary antibody HRP labeled (1:5000) for 1 hour at room temperature. After washing 5 × 5 minutes washes with TBS (pH 7.4), the membrane was developed with 4-chloro-1-naphtol substrate.

### Detection of Bt18 and GAPDH on CEM-SS cells via immunofluorescent staining

CEM-SS cells (1 × 10^6 ^cells/mL) were harvested by centrifugation at 130 × g for 5 minutes and washed 3 × 5 minutes with PBS. The cells were resuspended in PBS and smears were prepared by dropping the cell suspension onto poly-L-lysine coated slides. The smears were left for 1 hour to allow the cells to adhere to the slides. Next the cells were fixed in 4% paraformaldehyde for 15 minutes and washed with PBS briefly. Non specific binding was blocked using 5% BSA in PBS for 20 minutes. The smear was washed 3 × 5 minutes with PBS. After washing, the smear was incubated with 100 μg/mL of solubilised and activated Bt18 parasporal protein for 1.5 hours. The cells were treated with a mixture of two primary antibodies, rabbit polyclonal anti-Bt18 (1:10) and mouse monoclonal anti-GAPDH (1:1000, Abcam) for 1 hour at room temperature and labeled with a mixture of fluorescent dye-conjugated secondary antibodies, Texas Red-labeled anti-rabbit (1:200, Abcam) and Fluorescein (FITC)-labeled anti-mouse (1:128, Abcam) for 1 hour at room temperature in the dark. Finally, the cells were counterstained with 0.1 μg/mL Hoechst blue nuclear stain for 10 minutes. Negative controls included the omission of Bt18 parasporal protein, omission of primary antibodies (anti-Bt18 and anti-GAPDH antibody), and omission of secondary antibodies (Texas-Red and FITC).

### Confirmation of Bt18 binding to GAPDH via immunofluorescent staining

In order to confirm the binding of Bt18 parasporal protein to GAPDH in CEM-SS cells, the immunofluorescent staining protocol (as described above) was modified by incubating the slide with anti-GAPDH antibody (dilution 1: 1000, Abcam) for 1 hour before the step for adding 100 μg/mL Bt18 parasporal protein was taken. A similar slide without the anti-GAPDH antibody incubation step was prepared stimultaneously as positive control. Both slides were later viewed under fluorescence microscopy and compared for fluorescence intensity.

## Results

### Separation of Bt18 parasporal protein

**Figure 1:**
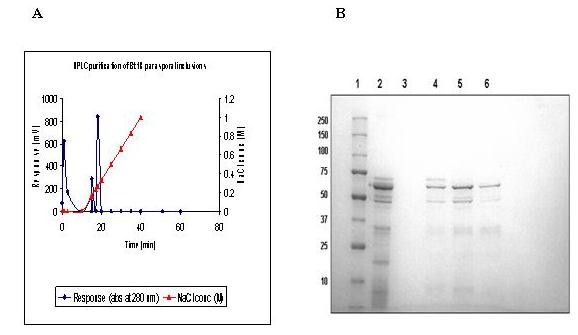
**A. HPLC Chromatogram of trypsin activated Bt18 parasporal protein**. Trypsin activated parasporal protein was applied to a Mono Q exchange column with 20 mM Piperazine buffer (pH 9.8). The column was set to run at 1.0 mL/min with the salt gradient (0-1 M NaCl). **B. SDS-PAGE profile of Mono Q purified trypsin activated Bt18 parasporal protein**. Coomassie Blue stained SDS 10% polyacrylamide gel. Lane 1: Molecular weight marker; Lane 2: Solubilised and activated Bt18 parasporal protein; Lane 3: HPLC Fraction 3; Lane 4: HPLC Fraction 4; Lane 5: HPLC Fraction 5; Lane 6: HPLC Fraction 6.

Upon solubilisation in sodium carbonate buffer (pH 10.5) and trypsin activation, Bt18 showed an abundant polypeptide band of 68-kDa and low molecular weight polypeptides ranging from 20-75-kDa (lane 2, Figure 1B). The 68-kDa parasporal protein was separated using Mono Q anion exchange column as shown in the chromatogram (Figure 1A). The 68-kDa Bt18 parasporal protein was eluted in the major peak (fraction 4-6) as evident in the SDS-PAGE gel (Figure 1B) with a reduction in the low molecular weight peptides. However, as the lower molecular weight polypeptides were still present in the purified fraction, the 68-kDa protein was eluted from the gel by passive elution, overnight. This gel eluted 68-kDa protein was used to raise antibody.

### Immunoblot assay to detect the binding of anti-Bt18 antibody to Bt18 parasporal protein

**Figure 2:**
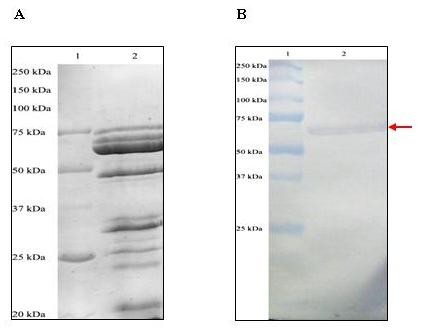
**Immunoblot assay to detect the binding of anti-Bt18 antibody to Bt18 parasporal protein; (A): SDS-PAGE gel; (B): western blot**. The detection of binding of anti-Bt18 antibody to Bt18 parasporal protein was performed as described in the methods section. Lane 1: Molecular weight marker; Lane 2: Bt18 parasporal protein. (Arrow indicates binding at approximately 68-kDa)

The immunoblot assay was performed to detect the binding of anti-Bt18 antibody to Bt18 parasporal protein. The immunoblot showed specific binding of the antibody to the parasporal protein at approximately 68-kDa as shown in Figure 2B, whereby Figure 2A is the corresponding SDS-PAGE profile of Bt18 parasporal protein. Interesting to note that cross-reactive binding was not observed on other polypeptides of Bt18 parasporal proteins, indicating the specificity of the antibody towards the 68-kDa parasporal protein. Sensitivity assay was also carried out using indirect ELISA method to evaluate the sensitivity of anti-Bt18 antibody on Bt18 parasporal protein. The result revealed that the antibody can detect as low as 25 ng/mL of Bt18 parasporal proteins.

### Immunostaining

**Figure 3:**
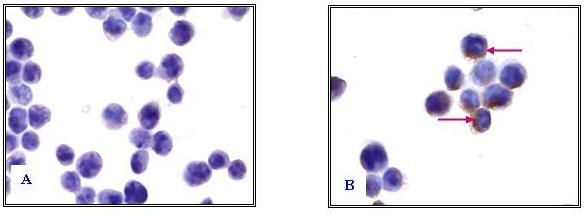
**Immunostaining of CEM-SS cells incubated with Bt18 parasporal protein**. The binding of Bt18 parasporal protein on CEM-SS cells was detected using immunostaining as described in the methods section. (A)- CEM-SS cells without Bt18 (negative control); (B)- CEM-SS cells incubated with Bt18. (1000× magnification) (Arrow pointing at the brownish ring formation).

Strong immunostain (brownish ring formation) were observed around the CEM-SS cells (Figure 3B) treated with Bt18 indicating possible localisation of Bt18 parasporal protein binding on plasma membrane periphery. The negative control cells (untreated) were observed as immuno-negative (Figure 3A) as no brown stains were observed.

### Receptor binding assay

**Figure 4:**
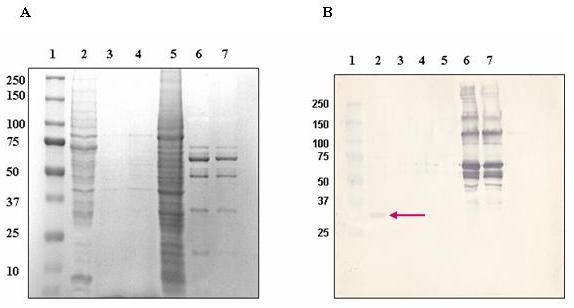
**Toxin overlay blot with Bt18 parasporal protein**. Toxin overlay blot was prepared as described in the methods section. (A): SDS-PAGE gel; (B): western blot. Lane 1: Molecular weight marker; Lane 2: CEM-SS cells lysate; Lane 3: Diluted membrane proteins (hydrophobic fraction); Lane 4: Undiluted membrane proteins (hydrophobic fraction); Lane 5: Hydrophilic fraction; Lane 6: Bt18 parasporal protein; 1 mg/mL; Lane 7: Bt18 parasporal protein; 0.5 mg/mL (Arrow pointing at the binding protein).

The membrane proteins used for the identification of putative receptor was harvested from CEM-SS cells using Mem-PER Eukaryotic Membrane Protein Extraction Reagent Kit. Harvested membrane proteins were subjected to detergent removal via dilution and dialysis using Slide-A-lyzer^® ^MINI Dialysis unit. The dialysate was subjected to SDS-PAGE to evaluate the protein component, however it was noted that many polypeptides were absent in the hydrophobic fraction (majority of membrane proteins should be in this fraction). We observed instead that most polypeptides were present in the hydrophilic fraction and not in the hydrophobic fraction, indicating that the harvest of the membrane proteins were not very efficient. After many and varied attempts, the detection of the putative receptor with membrane proteins harvested was not successful. For example, no binding was observed when the membrane proteins were incubated with the unpurified or purified Bt18 parasporal proteins. To overcome this difficulty, the putative receptor for Bt18 parasporal proteins was studied using freshly prepared CEM-SS cells lysate incubated with freshly activated Bt18 parasporal proteins. The binding protein for Bt18 parasporal protein was successfully identified as shown in Figure 4B (Lane 2). The binding protein had a molecular weight between 25-37-kDa (estimated 36-kDa). The 15 amino acids sequence obtained were **G-K-V-K-V-G-V-N-G-F-G-R-I-G-G (Gly-Lys-Val-Lys-Val-Gly-Val-Asn-Gly-Phe-Gly-Arg-Ilc-Gly-Gly)**. The amino acid sequence was analysed using the NCBI BLASTP; Swiss-Prot database and identified as **G3P-Human-Glyceraldehyde-3-phosphate-dehydrogenase **(GAPDH). The Alignment Score was 44.8 Bits and the expectation value (E-value) was 3 × 10^-5 ^[Swiss-Prot: P04406]. 14 of 15 amino acid sequence in the N-terminal of binding protein showed 100% match to the N-terminal of human GAPDH

### Detection of GAPDH in CEM-SS cell lysate via immunoblot assay

**Figure 5:**
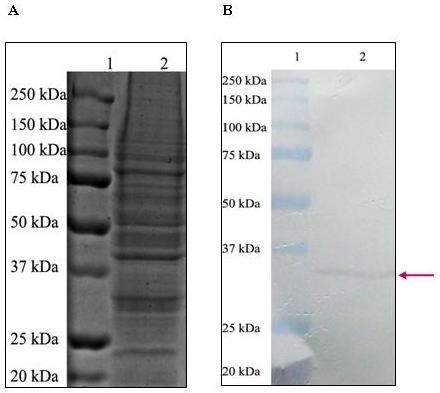
**Immunoblot assay to detect the expression GAPDH in CEM-SS cells crude cell lysate**. Immunoblot assay was carried out as mentioned in the methods section. (A): SDS-PAGE gel; (B): western blot. Lane 1: Molecular weight marker; Lane 2: CEM-SS cells crude lysate (5.0 mg/mL) (Arrow points to approximately 36-kDa, the estimated molecular weight of GAPDH).

The immunoblot showed a specific binding at a single polypeptide band of a molecular weight of 25-37 kDa (Figure 5B). It was concluded that the polypeptide is indeed GAPDH as the membrane was probed with anti-GAPDH antibody (Abcam). The molecular weight and gel position of the polypeptide corresponds with the binding protein of Bt18 parasporal protein in CEM-SS cells.

### Detection of Bt18 and GAPDH in CEM-SS cells via immunofluorescence staining

**Figure 6:**
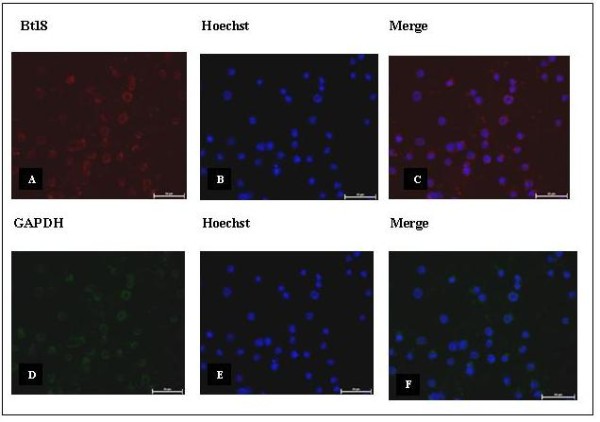
**Double immunofluorescence staining - detection of Bt18 binding to CEM-SS cells and detection of GAPDH expression in CEM-SS cells**. (400× magnification). (Scale bar = 50 μm). (A) Binding of Bt18 on CEM-SS cells detected using a Texas Red filter. (B) Nucleus of CEM-SS cells detected using a Hoechst filter. (C) Superimposed images of (A) and (B). (D) Detection of GAPDH expression CEM-SS cells using FITC filter. (E) Nucleus of CEM-SS cells detected using a Hoechst filter. (F) Superimposed images of (D) and (E).

**Figure 7:**
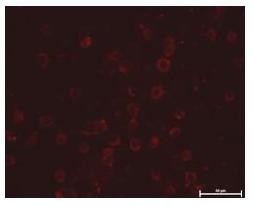
**Double immunofluorescence staining - colocalisation of Bt18 and GAPDH in CEM-SS cells, merged images of Figure 6(C) and Figure 6(F)**. Yellow fluorescence signals indicate co-localisation of Bt18 and GAPDH.

In the double immunofluorescent staining, anti-Bt18 antibody was used as a probe to detect the binding of Bt18 parasporal protein in the CEM-SS cells while anti-GAPDH antibody was used to detect the expression of GAPDH in the CEM-SS cells. Figure 6(C) showed red fluorescence signals around the cells indicating localisation of Bt18 parasporal protein in the plasma membrane of the cells. Figure 6(F) showed the expression of GAPDH in the CEM-SS cells proven by emission of green fluorescence signals around the cells as well. The double immunofluorescence image (Figure 7) suggested that Bt18 parasporal protein and GAPDH are co-localised in the plasma membrane of CEM-SS leukaemic cells.

### Confirmation of Bt18 binding to GAPDH via immunofluorescence staining

**Figure 8:**
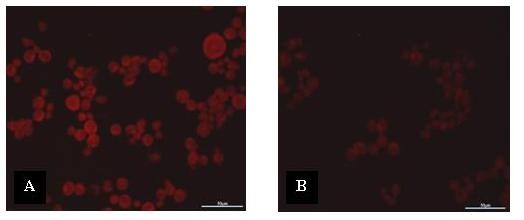
**Confirmation of Bt18 binding to GAPDH via immunofluorescence staining**. (Scale bar = 50 μm). (A) Without anti-GAPDH antibody incubation. (B) With anti-GAPDH antibody incubation.

To corroborate the possibility of Bt18 binding to GAPDH on plasma membrane of CEM-SS cells, anti-GAPDH antibody was used to block the GAPDH expressed on the plasma membrane. The cells were first incubated with anti-GAPDH antibody before treating with Bt18 parasporal protein. Figure 8A showed the image of the slide without anti-GAPDH antibody incubation and Figure 8B showed the image of the slide with anti-GAPDH antibody incubation before treating the cells with Bt18 parasporal protein. By using a red fluorescent to detect the binding of Bt18 to CEM-SS cells it was noted that there was a decrease in the intensity of fluorescence signals around the cells in the slide with anti-GAPDH antibody incubation (Figure 8B) as compared to the cells without anti-GAPDH antibody incubation (Figure 8A). The decrease in fluorescence intensity suggested that less Bt18 bound to the cells due to less available binding sites.

## Discussion

We began our study with purification of the Bt18 parasporal protein as it was a necessary step for raising antibodies against Bt18. When Bt18 parasporal protein was applied to a Mono Q 5/50 GL anion exchange column, this step led to the separation of a 68-kDa protein from the solubilised and activated parasporal protein. The cytotoxicity of the 68-kDa protein against CEM-SS cells was found to be reduced by 20% compared to the cytotoxicty exerted by the unseparated parasporal protein. This could possibily be due to the loss of polypeptides after separation, that were present in unseparated parasporal protein [14].

In order to localise the binding site for the Bt18 parasporal protein, we performed immunostaining of the CEM-SS cells with anti Bt18 antibodies. Antibody against Bt18 was successfully raised and detected in rabbit sera as early as three weeks after primary immunisation (data not shown). The sensitivity of the antibody raised was studied using immunoblot assay and strong binding was observed on the immunoblot developed with the anti-Bt18 antibody. Microscopic observation revealed that Bt18 parasporal protein was distributed at the cell periphery of CEM-SS (Figure 3) suggesting that the protein may bind to a receptor on the plasma membrane of cells. A study on parasporin-2 action on Hepatocyte cancer cells (HepG2) showed that the parasporal protein was detected at the cell periphery after incubation. Parasporin-2 was mostly distributed at the plasma membrane because the immunostaining pattern of these non-permeabilised cells was the same as the native distribution of cadherin, a cell-cell adhesion protein in the plasma membrane [15]. The study concluded that parasporin-2 was localised in the lipid raft of plasma membrane before and during the membrane damage and subsequently induces cell death. Similarly Bt18 parasporal protein was localised at the cell periphery during its activity suggesting that Bt18 parasporal protein and parasporin-2 may share similar mode of action.

To further elucidate the mode of action of Bt18 parasporal protein, we first determined the binding protein or putative receptor in CEM-SS cells during interaction with Bt18 parasporal protein. Interestingly Bt18 parasporal protein showed binding to a protein identified as GAPDH, which was present in the CEM-SS cell lysate. The interaction of Bt18 parasporal protein to GAPDH was further verified using purified 68-kDa protein [16]. The binding of a Bt parasporal protein to GAPDH has not been reported. To confirm Bt-GAPDH binding, we used double immunofluorescence microscopic studies. Red fluorescence signals around the cells indicating Bt18 is likely to bind on the plasma membrane of the cells (Figure 6C). The detection of GAPDH expression on CEM-SS cells (Figure 6F) showed green fluorescence signals seen around the cells indicating GAPDH is expressed on the plasma membrane of the cells as well. When both of the images were merged (Figure 7), a yellow fluorescence signal was produced around the cells, indicating co-localisation of Bt18 and GAPDH at the plasma membrane of the cells. To further confirm the binding of Bt18 parasporal protein to GAPDH in the leukaemic cells, we carried out immunostaining by first blocking GAPDH with anti-GAPDH antibody before incubating the cells with Bt18 parasporal protein. The results showed a reduction in the binding of Bt18 to the CEM-SS cells when compared with the slide without anti-GAPDH incubation (Figure 8). This further suggests that Bt18 binds to GAPDH on the cell membrane.

First discovered as one of the key enzymes involved in glycolysis, GAPDH exert several functions as diverse as apoptosis induction, receptor-associated kinase, tRNA export or DNA repair [17]. These functions have been linked to the various intracellular localisations of the enzyme, which has been found in the cytosol, nucleus, ER-golgi-vesiculae, mitochondria, as well as associated with the plasma membrane [18,19]. Interestingly, Xing *et al.*, (2004) [20] reported GAPDH as a target protein of the saframycin antiproliferative agents for leukaemia and tumour-derived cells, where it forms a ternary complex with saframycin-related compounds and DNA, inducing a toxic response in cells. A specific binding interaction occurred between GAPDH, duplex DNA, and several known members of the saframycin class of antiproliferative agents implicating a previously unknown molecular mechanism of anti-proliferative activity. This suggests that GAPDH may be a potential target for chemotherapeutic intervention. In a separate study, it is known that Bt18 causes cell death in CEM-SS via apoptosis, as demonstrated by Active caspase 3/7, Annexin V and TUNEL assays [13]. While in our study, we suggest that the Bt18-GAPDH binding contributes a significant role in the cell killing mechanism against CEM-SS cells. The above said data supports the suggestion that GAPDH is linked with apoptotic cell death.

Based on a qualitative analysis of the immunoblot and immunofluorescence results, it was suggested that GAPDH is a binding protein located on the plasma membrane of CEM-SS cells for Bt18 parasporal protein. Lee *et al.*, (2001) [1] suggested that the cytotoxic mechanisms of the anti-cancer parasporal proteins (parasporins) were similar to Cry proteins, which is dependent on binding to receptor(s) on the cell membranes. Previously, no reports have identified a putative receptor for *Bt *parasporins. However, it was reported that parasporin-2 was localised in the plasma membrane after incubation in Hep-G2 cells. They suggested that the final destination of the toxin for killing cells should be on the cell surface where the membrane damage occurs [2]. In a study on *Bacillus thuringiensis *subsp. *coreanensis *A1519 strain, ligand blotting analysis with cell membrane proteins of MOLT-4 and HeLa cells suggested that the bacterium was closely correlated with the presence of specific binding proteins with molecular sizes from 40 - 50-kDa in the cell membrane of MOLT-4. Thus, the *Bacillus thuringiensis *subsp. *coreanensis *A1519 strain may recognise and bind to a cell death inducing membrane protein of MOLT-4, setting the death signal [3].

Early studies have identified GAPDH as a membrane bound protein [4] and 60-70% of total erythrocyte GAPDH was found to be membrane associated [5]. In a study on the biosynthesis of GAPDH in prostate cancer, the presence of five isozymes of GAPDH in human malignant cells were reported while only four were detected in normal prostate tissue. This further suggested that multiple forms of GAPDH may have diverse roles in the cells [6]. Several studies reported an increase of GAPDH expression in cancer cell lines [7,8] and shows that it has roles in the neoplastic transformation of hepatocytes [9], tumor cell motility and metastasis in rat prostate adenocarcinoma tissue [10], and in the detoxification of cisplatin and doxorubicin in cancer cells [11]. A study using anti-sense oligodeoxynucleotide of the GAPDH gene inhibited cell proliferation and induced apoptosis in human cervical cancer cell lines [12]. These reports provide evidences for the existence of GAPDH isozymes and its functional diversities, which raises the possibility of GAPDH to function as a receptor in cancer cells for *Bt *parasporal proteins. The preferential toxicity of Bt18 to CEM-SS cells makes Bt18 a possible chemotherapeutic agent alone or as a synergist with current anticancer agents to enhance its cytotoxicity against cancer cells. Therefore, identification of a receptor would provide insights to the mechanism of action of Bt18 parasporal protein which is crucial in the pharmacological understanding of Bt18.

It is noteworthy that GAPDH has been reported to be expressed on the surface of macrophage membrane and reported to function as novel transferrin receptor [33]. FACS analysis of monoclonal anti-GAPDH antibody stained J774 mouse and human macrophage cell line demonstrated that these cells express GAPDH on the membrane surface. The presence of GAPDH on the outer surface of intact J774 cell membrane was further confirmed by immunolabelling followed by transmission and electron microscopy. Interestingly the study also indicated that mammalian GAPDH showed interaction with human holo-transferin. Transferrin colocalises with cell surface GAPDH as shown by confocal microscopy of double immunoflourescence staining of intact J774 cells. GAPDH-transferrin interaction was further proved using *invitro *ELISA assay and FRET analysis. GAPDH was also noted to play a role in the induction of apoptosis by nuclear translocation of endogenous GAPDH. Over expressed GAPDH that is translocated in the nucleus preceding DNA damage robustly induced apoptotic death [34]. Furthermore, in apoptotic cells, GAPDH expression is three times higher than in non-apoptotic cells. This could probably related to the activity of GAPDH as a DNA repair enzyme or as a nuclear carrier for pro-apoptotic molecules [35] These findings suggest that there may be a role for GAPDH in the mode of action for Bt18 parasporal protein. It is interesting to note that Bt18 parasporal protein act like parasporins, as these proteins are non-haemolytic and capable of preferentially killing leukaemic T cells. Thus, binding of Bt18 parasporal protein to GAPDH is a significant finding as literature shows that there have been limited studies on identification of a binding protein for parasporins in cancer cells.

## Conclusion

We conclude that there is a binding protein in CEM-SS cell for Bt18 parasporal protein. G3P-Human-Glyceraldehyde-3-phosphate dehydrogenase (GAPDH) was identified as the binding protein of cytotoxic Bt18 parasporal protein. The findings in this study warrant further investigations to determine the different isozymes present in leukaemic cells compared to normal T-lymphocytes. Future gene therapy against cancer specific GAPDH isozymes might be another form of treatment in cancer.

## Competing interests

The authors declare that they have no competing interests.

## Authors' contributions

KK participated in experimental design, data acquisition, interpretation, writing and editing of this manuscript. JEAK participated in data acquisition and interpretation. SMM participated in experimental design, data interpretation and editing of the manuscript. VDN contributed to experimental design, data interpretation, editing and submission of this manuscript. All authors read and approved the final manuscript.
